# Rare Case of Gallbladder Neuroendocrine Carcinoma

**DOI:** 10.7759/cureus.28531

**Published:** 2022-08-29

**Authors:** Andrew T Rennie, Steven L Halbreich

**Affiliations:** 1 Clinical Sciences, Florida State University College of Medicine, Tallahassee, USA; 2 Surgery, Florida State University College of Medicine Sarasota Regional Campus, Sarasota, USA

**Keywords:** gi oncology, neuroendocrine carcinoma (nec), gallbladder neuroendocrine carcinoma, gi tract, gallbladder neoplasm, neuroendocrine

## Abstract

Neuroendocrine tumors (NET) are very uncommon, though when seen, they are typically found within the gastrointestinal tract. Rarely NETs can be seen within the lumen of the gallbladder. An 83-year-old woman presented to our hospital with vague abdominal pain and distention. She was found to have a mass within her gallbladder on imaging, and she was treated with surgical resection. Pathology revealed a gallbladder NET with lymphovascular involvement. NETs of the gallbladder remain a rare occurrence with a limited number of previous case reports.

## Introduction

Neuroendocrine tumors (NET) are a form of neoplasia that has previously been described with an incidence of 5.25 per 100,000 [1]. NETs of the gastrointestinal tract have been previously well described, commonly occurring in the jejunum, anus, appendix, and pancreas [2]. While the incidence of NET of the gallbladder is far less common, comprising only 0.5% of all NETs [3]. The gallbladder is relatively devoid of neuroendocrine cells within the gallbladder mucosa, except for the gallbladder neck, which helps to explain the extremely low incidence [4,5]. There have been previous cases reported of NETs of the gallbladder [2,6-9]. However, the number of cases in the literature remains low, and the origin of these tumors is not well understood. We report a case of neuroendocrine carcinoma originating in the gallbladder.

## Case presentation

An 83-year-old Spanish-speaking female with a past medical history of non-insulin-dependent diabetes mellitus and hypertension presented for evaluation. She presented to our hospital as a transfer from a local community hospital. She reported a history of intermittent sharp right upper quadrant (RUQ) pain and diffuse lower pelvic pain that had been worsening over the past six months. Six months ago, when the pain initially started, she was diagnosed and treated for a UTI. She denied any recent fever, chills, unintentional weight loss, nausea, vomiting, constipation, diarrhea, hematuria, vaginal bleeding, dysuria, melena, or hematochezia. Additionally, the patient had never had any screening mammograms, Pap smears, or colonoscopies.

**Figure 1:**
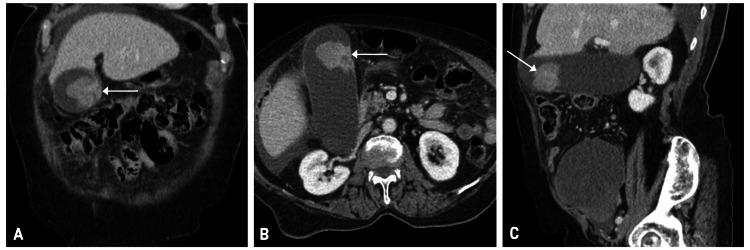
(A) Coronal, (B) Axial, and (C) sagittal views of initial CT scan revealing a mass (white arrows) within the gallbladder.

Before transfer, a CT abdomen was done, which showed a distended gallbladder with an intraluminal 3 x 3.1 x 3.7 cm gallbladder mass (Figure 1). The common bile duct measured up to 1 cm in diameter but appeared to taper smoothly. Additionally, there was a large pelvic mass inseparable from the fundal region of the uterus extending cephalad to the right of the midline, which measured 11 x 12.6 x 10 cm. Due to these findings, the patient was transferred for a higher level of care.

**Figure 2:**
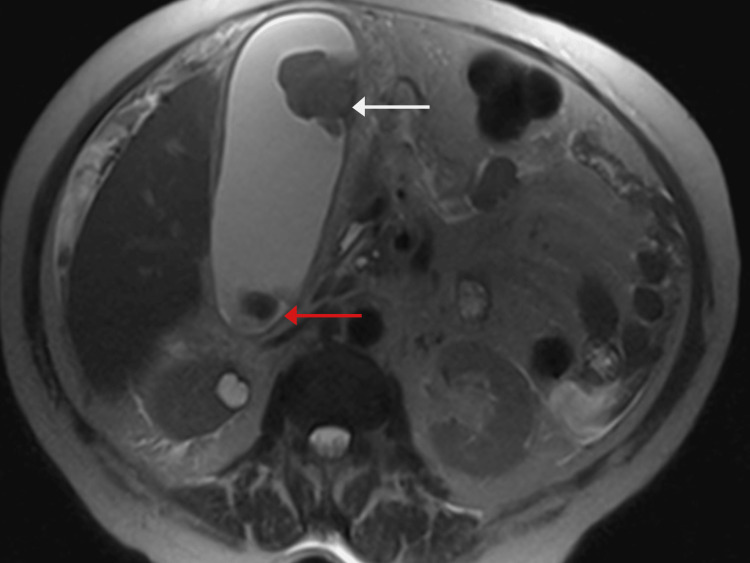
MRI demonstrating a large mass within the gallbladder (white arrow) and one of the two gallstones (red arrow) identified.

On examination, the patient was a well-appearing Hispanic female in no acute distress. Her sclerae were anicteric bilaterally. On examination of the abdomen, there was mild distention with diffuse abdominal tenderness, though worse in the right upper quadrant (RUQ). There was a palpable mass present in the RUQ with a negative Murphy sign. The rest of the exam was benign. Laboratory studies were completed, and the CA-125 level was found to be 139 units/mL (Table 1). Magnetic resonance imaging (MRI) with the contrast of the abdomen showed a distended gallbladder with abnormal gallbladder wall thickening and a soft tissue mass in the gallbladder fundus measuring 2.9 x 2.6 x 3.3 cm with broad-based attachment to the gallbladder wall (Figure 2). There was adjacent gallbladder wall thickening with heterogeneous enhancement and a high suspicion for malignancy. Additionally, two gallstones were noted to be present in the gallbladder neck. Pelvic ultrasound revealed a bulky heterogeneous solid 13 cm soft tissue mass in the midline pelvis anterior to the uterus. There was also a small number of ascites, and the ovaries could not be well visualized. CT of the thorax was negative for evidence of metastasis.

**Table 1:** Initial lab values obtained on patient presentation INR: international normalized ratio, GFR: glomerular filtration rate, AST: aspartate aminotransferase, ALT: alanine transaminase, CA-125: cancer antigen 125

Result	Reference Range
Complete Blood Count W Differential		
White Blood Cells	10.2	4.5 – 11.0
Red Blood Cells	3.86	4.0 – 5.2
Hemoglobin	11.9	10.9 – 15.5
Hematocrit	34.7	35.0 – 47.0%
Mean Corpuscular Volume	89.9	80.0 – 100.0
Platelets	199	150 – 400
Lymphocytes	14	15 – 49%
Segs	74	29 – 66%
Monocytes	11	2 – 14%
Eosinophils	1	0 – 5%
Basophils	0	0 – 2%
INR	1.13	0.88 – 1.13
Chemistries		
Glucose	131	70 – 100
Sodium	136	131 – 145
Potassium	3.4	3.5 – 5.1
Chloride	103	98 – 110
Bicarbonate	28	21 – 32
Blood Urea Nitrogen	16	8 – 23
Creatinine (serum)	0.66	0.55 – 1.02
Estimated GFR	>60	>60
Calcium	8.5	8.3 – 9.9
Liver Profile		
Total Bilirubin	0.9	0.2 – 1.3
Alkaline Phosphatase	98	33 – 149
AST	17	15 – 37
ALT	20	13 – 56
Total Protein (serum)	6.0	6.4 – 8.3
Albumin	2.3	3.2 – 4.8
Lipase	42	73 – 393
CA-125	139	0 – 35

The patient was treated with oxycodone for her pain and piperacillin/tazobactam for possible cholecystitis. She was scheduled for a robotic-assisted bilateral salpingo-oophorectomy and possible hysterectomy with gynecologic oncology and a robotic-assisted cholecystectomy with lymph node dissection with general surgery.

The patient successfully underwent robotic-assisted bilateral salpingo-oophorectomy. A frozen section was completed showing a specimen consistent with an ovarian fibroma. A robotic-assisted cholecystectomy was attempted, but due to the level of inflammation and complexity, it was converted to open cholecystectomy. The gallbladder and a large discolored cystic duct lymph node were sent for a frozen section, which revealed neuroendocrine carcinoma versus lymphoma. There was no evidence of adenocarcinoma. Only one lymph node was removed.

**Figure 3:**
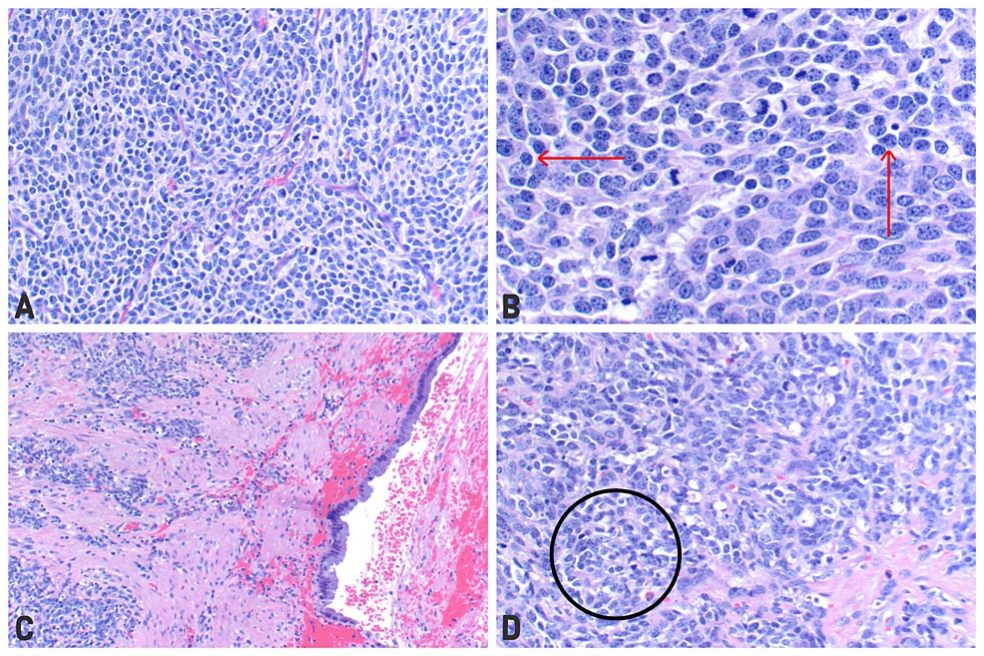
H&E staining of the resected lymph node (A and B) and the gallbladder wall (C and D). (B) and (D) demonstrate areas of hypercellular proliferation (black circle) with nuclear atypia (red arrows).

In the immediate postoperative period, the patient did very well. Final surgical pathology was reported on postoperative day (POD) #2. The final pathology revealed high-grade neuroendocrine carcinoma of the gallbladder with extensive necrosis, extending 5.0 cm into the muscularis and perimuscular tissue. There was no involvement of the serosa, liver, or cystic duct. However, there was a lymphovascular invasion present. Microscopic analysis and staining revealed neoplastic cells with the following immunophenotype: synaptophysin+, CD56+, AE1/AE3+, and CAM5.2+ (Figure 3). Immunohistochemical staining demonstrated a Ki67 proliferation rate of 70%. Due to these findings, her cancer stage was neuroendocrine carcinoma pT2b pN1 of 1. Inpatient hematology and oncology were consulted, who recommended outpatient follow-up with evaluation for possible adjuvant chemotherapy. The patient was discharged home on POD #3.

She was seen three weeks after discharge by a hematology and oncology specialist who obtained a PET/CT scan which showed nonspecific diffuse lymphadenopathy in the supraclavicular, mediastinal, and porta hepatis regions. The decision was made to start carboplatin and etoposide as adjuvant therapy. Her dose was subsequently reduced two weeks later due to nausea and fatigue. At her next visit, the patient was continued on her reduced dose of carboplatin/etoposide, and atezolizumab was added to her treatment regimen. Additionally, radiation oncology was consulted for possible concurrent radiation therapy. The patient, at this time, has been doing well with her therapies.

## Discussion

Gallbladder NETs remain relatively rare within the clinical setting. Previous studies have sought to determine the incidence of such tumors. Duffy et al. previously studied the incidence of gallbladder NETs at Memorial Sloan-Kettering Cancer Center and found that 435 patients between the years 1995 and 2005 were diagnosed with gallbladder carcinoma, and three percent of those patients had a NET [6]. Chen et al. described similar findings in their analysis of 387 patients with gallbladder carcinoma, finding 2.2% of patients to have a NET [2].

One of the challenges facing patients with gallbladder NETs is their propensity to present late in the disease process. Many patients are asymptomatic and often present with non-specific symptoms. This is very similar to our patient, whose pain was most likely from cholelithiasis resulting in chronic cholecystitis. NETs possess the ability to produce hormones such as serotonin, chromogranin-A, and gastrin, thus, there is a possibility for hormone-related side effects such as flushing, diarrhea, and coughing. However, these symptoms are non-specific, and less than one percent of patients with NETs present with this constellation of symptoms, called carcinoid syndrome [10].

The pathogenesis of gallbladder NETs is not entirely understood; however, it is believed that older female patients are at increased risk [7]. While most cases occur sporadically, there is thought to be an increased risk in those with chronic inflammation from pancreatitis or cholecystitis [1]. It is believed that this chronic inflammation can cause intestinal or gastric metaplasia of the gallbladder epithelial cells [8]. This hypothesis is supported by our case of an older female with pathologic and surgical signs of chronic cholecystitis.

Gallbladder NETs are classified based on the World Health Organization, which include grades G1, G2, and G3. G1 and G2 tumors are well differentiated, and G3 tumors are poorly differentiated, termed neuroendocrine carcinoma (NEC). The classifications are further described by the Ki-67 proliferation index. Grade G1 lesions have a Ki-67 index <3%, G2 lesions have a Ki-67 index of 3%-20%, and G3 lesions with a Ki-67 index >20% [11]. Thus, this patient had a G3 neuroendocrine carcinoma.

The prognosis for gallbladder NECs remains relatively poor due to the often late clinical presentation and the high propensity for malignancy. Chen et al. found their 1-, 2-, and 3-year survival rates to be 20%, 10%, and 0%, respectively [2]. They additionally found a statistically significant difference in survival rates of patients with gallbladder NETs to patients with gallbladder adenocarcinoma, partially due to the tumor’s rapid progression and lymphatic metastases.

Treatment remains difficult for patients diagnosed with gallbladder NETs with the most preferred and chosen treatment being surgical resection. The difficulty and complexity of the resection increase with lymphovascular involvement or metastases. Because most patients are diagnosed at a late stage, surgical resection is often not a viable option. For patients who are candidates for surgery, adjuvant chemotherapy is often employed following resection. Elahi et al. described an average survival time of 46 months when patients received adjuvant chemotherapy with a combination of gemcitabine, cis-platinum, docetaxel, and sunitinib [9]. This suggests a benefit to patients who receive combination chemotherapy and surgical resection. One prior study discussed a patient who received neoadjuvant chemotherapy; however, that patient had concurrent small cell lung cancer [12]. Although, larger patient populations and further analysis of clinical outcomes are needed to guide future treatment.

## Conclusions

Gallbladder NECs remain a rare and clinically devastating diagnosis. As there are no classic clinical findings and the presence of carcinoid syndrome occurs in such few patients, the patient often presents late in the disease course. This leads to further difficulty in treatment and poor clinical outcomes. Initial studies have shown improved results when combining surgical resection, chemotherapy, and possibly radiotherapy. However, with such a limited number of cases, further studies are needed to determine how best to treat these tumors.
